# Belimumab 10 years later: how drug positioning has changed

**DOI:** 10.1007/s12026-024-09543-z

**Published:** 2024-10-02

**Authors:** Fulvia Ceccarelli, Francesco Natalucci, Claudia Ciancarella, Licia Picciariello, Valeria Moretti, Francesca Romana Spinelli, Cristiano Alessandri, Fabrizio Conti

**Affiliations:** Lupus Clinic, Rheumatology Unit, Department of Clinical, Internal, Anesthesiological and Cardiovascular Sciences, Sapienza University of Rome, Viale del Policlinico 155, 00161 Rome, Italy

**Keywords:** Systemic lupus erythematous, Treatment, Biological drugs, Belimumab

## Abstract

We analysed the change in the positioning of belimumab (BLM) in systemic lupus erythematosus (SLE) treatment in the first decade of real-life use, by providing data about patients treated by this biological drug in the Sapienza Lupus Cohort. We evaluated SLE patients treated by BLM according to the current clinical practice. Data of each patient were collected, focusing on previous and concomitant treatments. Finally, the drug retention rate was assessed. Since August 2013, 138 SLE patients started BLM (M/F 7/131; median age 49 years, IQR 13.25; median disease duration 214 months, IQR 180). To evaluate the change in BLM positioning, we divided patients according to the date of starting treatment as below: patients treated from 2013 to 2018 (period 1) and those treated since 2019 to date (period 2). Indeed, the median number of previous immunosuppressant drugs was significantly higher in patients treated in period 1 [3 (IQR 1.25) *versus* 1 (IQR 1.75), *p* = 0.0002]. Furthermore, 15.9% of patients treated in period 2 were not previously treated by immunosuppressant drugs, compared with 5.2% in period 1 (*p* = 0.01). Finally, the 24-month drug survival was significantly higher in patients previously treated with ≤ 1 immunosuppressant drug in comparison with those treated with ≥ 2 drugs (69.1% versus 43.4%, *p* = 0.0097, HR 0.49; 95% CI 0.27–0.88). Our data clearly described the progressive anticipation of BLM prescription in the first 10 years of clinical practice, underlining as choosing earlier biological agents could positively influence the drug retention rate.

## Introduction

Systemic lupus erythematosus (SLE) is a chronic autoimmune disease, characterized by a great heterogeneity in terms of clinical phenotypes [1].

As clearly stated in the treat-to-target recommendations, SLE management aims at controlling disease activity, preventing the organ damage, and improving quality of life [2]. Indeed, damage is a multifactorial phenomenon and its development is driven by several factors [3]. The key role of disease activity has been clearly demonstrated by the evidence that remission achievement could significantly reduce the progression of damage [4]. However, a relevant contribution is made by the drugs administered to control disease activity. In particular, glucocorticoids (GCs) play a substantial role in determining the damage and their sparing represents a key step in modifying damage course [3].

In the past decade, we have witnessed a major change in the scenario of SLE treatment [5]. After many attempts by various randomised controlled trials, a biologic drug has been granted the indication for the treatment of patients with active disease despite standard of care treatment. This is belimumab (BLM), a monoclonal antibody targeting BLyS. Two randomised controlled trials ( BLISS-52 and BLISS-76) demonstrated the efficacy and safety of BLM in non-renal SLE patients, while more recently BLM was approved to treat lupus nephritis patients [6–8].

The wide use of this drug in real-life contexts led to the availability of extensive data, confirming the effectiveness of BLM outside of registration trials [9, 10].

In 2019, BLM appeared for the first time in the EULAR recommendations for SLE management [11]. At that time, the drug was recommended in patients with extra-renal disease inadequately controlled by first-line treatment, generally including a combination of hydroxychloroquine (HCQ) and GCs with or without immunosuppressant agents. Furthermore, the drug should be considered in patients unable to reduce GC dosage lower than 7.5 mg/daily [11].

The BLM effectiveness confirmed by its growing use in a real-life context has been received by the 2023 updated recommendations [12]. Accordingly, the use of biological drugs is recommended in patients not responding to HCQ, alone or in combination with GCs, or in patients unable to reduce GCs below acceptable doses for chronic use. Thus, these new recommendations suggest that the prescription of biological drugs could be anticipated at the same position as immunosuppressive drugs [12]. Furthermore, another fundamental change concerns the acceptable maintenance GC dosage, now reduced to 5 mg/day, with the suggestion of withdrawal when possible [12].

In the present study, we evaluated the change in the BLM prescription during the first 10 years of use in a real-life context, with a particular focus on the drug positioning. For this purpose, we reviewed data about SLE patients treated with BLM at the Sapienza Lupus Cohort since 2013, when the drug was approved to treat SLE patients in Italy.

## Methods

For the present analysis, we evaluated all the SLE patients (ACR criteria 1997, ACR/EULAR 2019 criteria) [13, 14] treated with BLM according to the current clinical practice since August 2013. All the patients were followed at the Lupus Clinic of Sapienza University of Rome (*Sapienza Lupus Cohort*).

The clinical, demographic, and laboratory data of each patient were collected in a standardised computer-filled form, including the date of diagnosis, disease-related manifestations, immunological features, and previous and ongoing treatments. Disease activity was assessed by Systemic Lupus Erythematosus Activity Disease Index-2 K (SLEDAI-2 K) and chronic damage by SLICC Damage Index (SDI) [15, 16].

BLM was added to background therapy according to the indication of the Italian Agency of Drug and was administrated intravenously at 10 mg/kg on days 1, 14, 28, and then every 28 days, or subcutaneously at 200 mg/weekly. All the patients were assessed at baseline (T0) and after 1, 3, 6, 12, and 24 months of follow-up (T0, T1, T3, T6, T12, and T24, respectively).

This study was conducted according to the Declaration of Helsinki statements. The Ethical Committee of AOU Policlinico Umberto I, Rome, approved the study protocol. All patients signed the informed consent.

### Statistical analysis

Version 9.0 of the GraphPad statistical package was used for statistical analysis. Normally distributed variables were summarised using the mean ± SD, and non-normally distributed variables by the median and range. Frequencies were expressed by percentage. Wilcoxon’s matched pairs test and paired *t*-test were performed accordingly. Univariate comparisons between nominal variables were calculated using the *χ*2 test or Fisher’s exact test where appropriate. Spearman’s test was used to assess the correlations. The Kaplan–Meier analysis was applied to evaluate survival treatment (Mantel-Cox analysis).

Two-tailed *p*-values were reported, and *p*-values < 0.05 were considered statistically significant.

## Results

Since August 2013, 138 SLE patients started BLM treatment (M/F 7/131; median age 49 years, IQR 13.25; median disease duration 214 months, IQR 180) due to inadequate disease control. Table 1 reported the main clinical and laboratory features of SLE patients at baseline.

**Table 1:** Clinical and laboratory features of patients at baseline

Clinical features	*N* (%)
Musculoskeletal involvement	92 (66.7)
Cutaneous involvement	34 (24.6)
Haematological involvement	14 (10.1)
Lupus nephritis	4 (2.9)
Serositis	3 (2.2)
Fever	8 (57.9)
Laboratory features	*N* (%)
Anti-dsDNA	122 (88.4)
C3 low	90 (65.2)
C4 low	86 (62.3)

The most relevant disease-related manifestation at the beginning of BLM therapy, which can be considered the indication for treatment, was joint involvement (83 patients, 60.1%), followed by skin manifestations (28 patients, 20.3%).

Regarding the drug administration route, 52 patients (37.7%) were treated intravenously and the remaining 86 (62.3%) subcutaneously. SLE patients treated intravenously had a significantly longer median disease duration than those treated subcutaneously [250 (IQR 126) months *versus* 166 (IQR 216) months, *p* = 0.001].

**Fig. 1:**
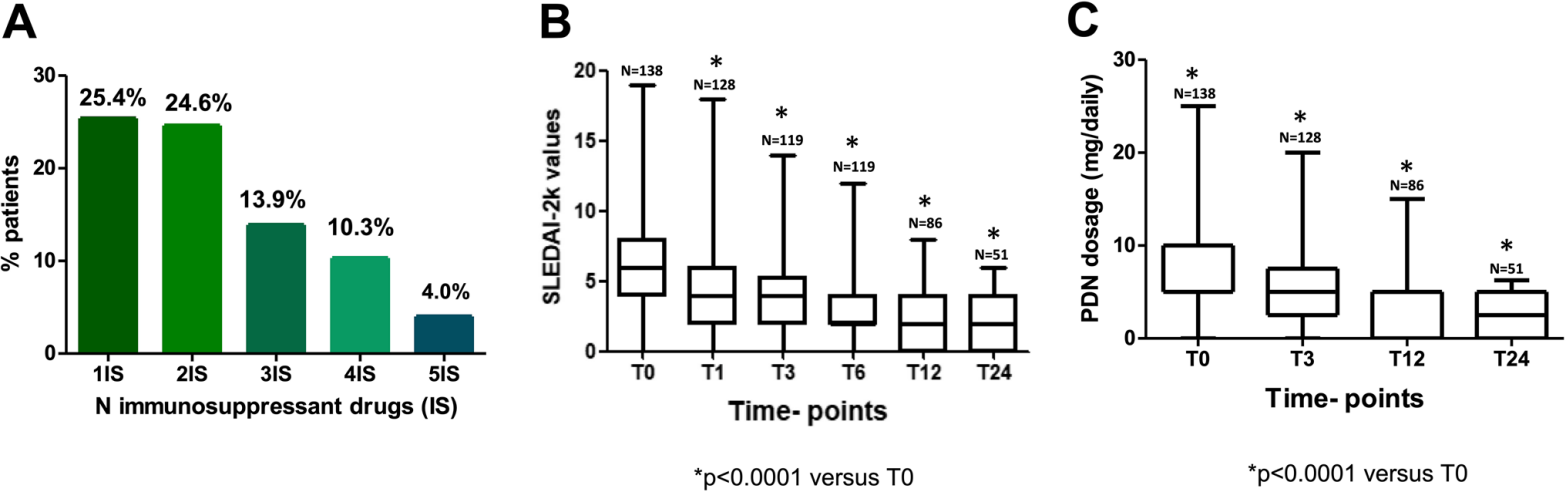
**A** Distribution of previously used immunosuppressant drugs. **B** SLEDAI-2 k values in the different time-points represented by box and whisker graph. **C** Glucocorticoid dosage (PDN mg/daily) in the different time-points represented by box and whisker graph

Concerning the previous immunosuppressant drugs, 84.1% of patients were treated with at least one immunosuppressant agent before starting BLM. The distribution of the number of previously taken immunosuppressant drugs has been reported in Fig. 1A.

The use of ≥ 2 immunosuppressant drugs before BLM administration was associated with acute cutaneous lupus (*p* = 0.01, *r* = 0.21, 95% CI 0.03–0.39) and with higher SLEDAI-2 k values at baseline (*p* = 0.02, *r* = 0.20, 95% CI 0.01–0.38). Furthermore, the median number of previous immunosuppressant drugs was significantly higher in patients treated with intravenous compared to those treated with subcutaneous BLM [3 (IQR 1.25) *versus* 1 (IQR 1), *p* < 0.0001].

At the BLM therapy beginning, 107 patients (77.5%) were taking HCQ while 81 (58.7%) were taking immunosuppressive drugs (mycophenolate mofetil 45.7%; azathioprine 28.4%, methotrexate 13.6%, cyclosporine A 11.1%, leflunomide 1.2%).

SLE patients were treated with BLM for a mean period of 24.0 months (SD 23.7; median 15.0; IQR 25.7). During the follow-up, we observed a significant reduction in SLEDAI-2 k values already after 1 month of treatment (*p* = 0.001), which was maintained at all subsequent time points (*p* < 0.0001) (Fig. 1B). Likewise, we observed a significant reduction in the median daily prednisone (PDN) dosage, again at T1 (*p* < 0.0001) and confirmed in all the subsequent time points (*p* < 0.0001, Fig. 1C). In addition, at baseline 14.5% of patients were not taking PDN; this percentage increased at T1 to 20.2%, reaching 40.0% after 24 months of treatment (*p* < 0.0001). No significant changes were observed in mean SDI values during the follow-up (T0: mean ± SD 0.76 ± 1.10; T12: mean ± SD 0.91 ± 1.19; T24: mean ± SD 0.91 ± 1.39; *p* = not significant). It should be mentioned that 42 SLE patients despite being in treatment had not yet reached 24 months of follow-up.

We evaluated the drug survival by the Kaplan–Meier analysis, revealing a survival rate equal to 75.5% at T12 and 59.5% at T24. This rate was significantly higher at T12 for subcutaneous compared to intravenous administration (67.1% *versus* 49.5%, *p* = 0.01, Mantel-Cox analysis). BLM withdrawal was associated with significantly higher disease duration (*p* = 0.02, *r* = 0.19, 95% CI 0.02–0.36) and with a higher number of previous immunosuppressant drugs (*p* = 0.003, *r* = 0.258, 95% CI 0.08–0.41). About the reasons for BLM discontinuation, the majority of patients stopped BLM due to adverse events (*N* = 16, 11.6%) and loss of efficacy (*N* = 15, 10.9%), seven patients (5.1%) were lost to follow-up (five of them due to change of reference center), six patients (4.3%) due to primary inefficacy, two patients (1.4%) for poor adherence to treatment, and two (1.4%) for diagnosis of cancer.

In order to evaluate the change in BLM positioning during the observation period, we divided patients according to the date of starting treatment as below: patients treated from 2013 to 2018 (period 1, *N* = 44) and those treated since 2019 to date (period 2, *N* = 94).

The comparison between these subgroups of SLE patients revealed a change in the distribution of the disease-related features leading to the introduction of BLM. In detail, in period 1, 72.7% of patients were treated for joint involvement, followed by skin manifestations (15.6%) and haematological features (4.5%). In period 2, we observed a reduction in the proportion of patients treated for joint involvement (54%) and the increase in those treated due to skin and haematological features (22.3% and 7.4%, respectively); furthermore, other disease-related manifestations, such as lupus nephritis and serositis was considered for BLM administration (4.2% and 3.2%, respectively).

Indeed, we observed a median number of previous immunosuppressant drugs significantly higher in patients treated in period 1 compared with those treated in period 2 [3 (IQR 1.25) versus 1 (IQR 1.75), *p* = 0,0002]. Furthermore, 15.9% of patients treated in period 2 were not previously treated by immunosuppressant drugs, compared with 5.2% in period 1 (*p* = 0.01).

**Fig. 2:**
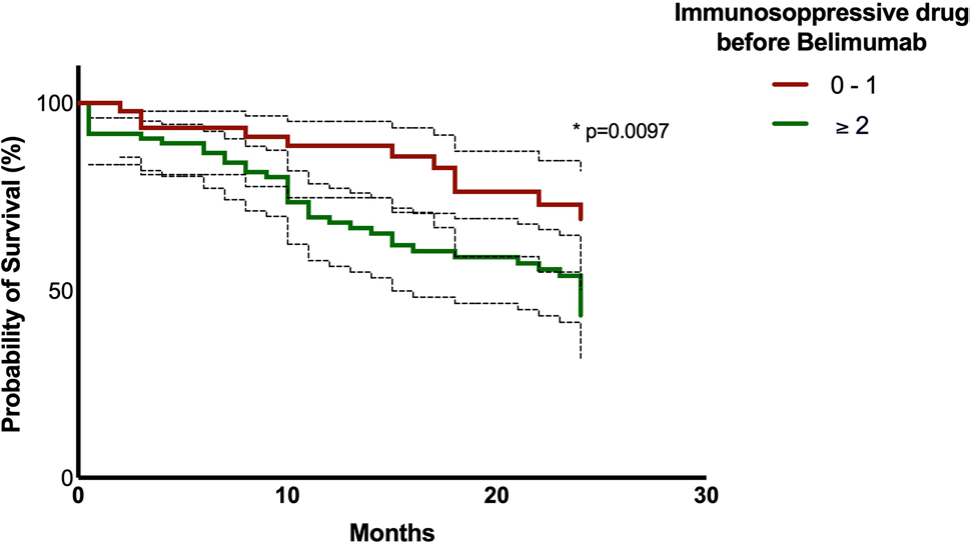
Survival of BLM treatment according to the number of previous immunosuppressant drugs

Accordingly, we evaluated 24-month BLM survival based on to the number of previous immunosuppressant drugs. As reported in Fig. 2, the drug survival at 24 months was significantly higher in patients previously treated with ≤ 1 immunosuppressant drug in comparison with those treated with ≥ 2 immunosuppressant drugs (69.1% versus 43.4%, *p* = 0.0097, HR 0.49; 95% CI 0.27–0.88, Mantel-Cox analysis).

Furthermore, patients previously treated with ≤ 1 immunosuppressant drug were more likely to stop GC treatment. Indeed, the proportion of SLE subjects stopping GCs was higher in those treated with ≤ 1 immunosuppressant drug compared with those treated with ≥ 2 immunosuppressant drugs, reaching a significant difference at 24 months of treatment (T3 30.2% *versus* 12.1%, *p* = 0.04; T12 40.5% *versus* 19.5%, *p* = 0.05; T24 73.3% *versus* 28.0%, *p* = 0.008).

## Discussion

The therapeutic algorithm for the management of SLE patients has been substantially modified by the latest recommendations, which clearly suggest the anticipation of biological agents in the same position as immunosuppressive drugs [12]. This represents a major innovation for the treatment of SLE patients and is certainly derived from the efficacy and safety data of randomised controlled trials and by the wide use of BLM in a routine scenario [17].

Indeed, the present analysis aims to provide information about the change in BLM positioning in the first 10 years of use in a real-life scenario. The availability of such a long observation period allowed to clearly demonstrate the progressive anticipation of BLM prescription in SLE patients. Indeed, the median number of previous immunosuppressant drugs has significantly decreased in the last 5 years (2019–2023) compared to what was registered in the first 5 years of observation (2013–2018). In parallel, we observed the increase in the proportion of patients not previously treated with any immunosuppressant before starting BLM: indeed, this proportion has reached about 15% in the 2019–2023 time period.

These results suggest that, whether in the first 5 years BLM was considered one of the last therapeutic alternatives for multi-resistant patients with long-standing disease, the choice of BLM was gradually anticipated until its placement alongside immunosuppressant drugs, and thus after first-line treatment. This is probably due to the large amount of data showing the effectiveness of BLM in a real-life setting, together with the steroid-sparing effect and long-term disease control. A significant contribution on this field has been provided by the multicentre Italian register BeRLiSS (*Belimumab in Real Life Setting Study*). In the study published in 2018, the authors reported a drug survival equal to 76.9% and 67.1% at 12 and 24 months, respectively, resulting from the BLM effectiveness and its good safety profile [9]. Similar results have been reported by the OBSErve program, including patients from Argentina, Canada, Germany, Spain, Switzerland, and the USA [17]. These results overlap with what was observed in our cohort, in which the BLM survival rate was equal to 75% at 12 months and about 60% at 24 months.

Certainly, the introduction of an easier subcutaneous route of administration facilitated the use of BLM. As reported in the literature, the majority of patients prefer subcutaneous administration underlining the importance of not losing working days, avoiding hospital visits, and being autonomous and comfortable at home [18].

We observed a significantly higher number of previous immunosuppressant drugs in patients treated with intravenous compared to those treated with subcutaneous BLM. It must be considered that subcutaneous administration has been introduced more recently than intravenous administration; consequently, the more recent availability and convenience of subcutaneous administration could certainly influence earlier BLM prescription in these patients.

Moving from these results, we evaluated BLM survival rate according to the number of previous immunosuppressant drugs. Indeed, patients previously treated with ≤ 1 drug showed a significantly higher survival rate compared with those treated with > 2 drugs. This result goes hand in hand with the significant association between BLM withdrawal and a higher number of previous immunosuppressant drugs. In parallel, the drug discontinuation was associated with longer disease duration.

This data underlines as early BLM introduction could positively influence the drug retention rate, that was widely recognized as the combination of the efficacy and safety of the considered drug.

Moreover, the early use of BLM seems to facilitate the discontinuation of GCs. In fact, the proportion of patients able to stop GCs was higher in patients previously treated with a lower number of 1 immunosuppressant drug. This aspect is certainly relevant in the light of the strict relationship between GCs and organ damage development. Indeed, the early introduction of BLM, by increasing the possibility to withdrawn GCs could play a pivotal role in preventing damage.

In conclusion, we provide new information about BLM use, focusing on the change of its positioning in the treatment of SLE patients. Our data clearly describe the progressive anticipation of BLM prescription in the first 10 years of clinical practice, underlining how the anticipation of biological agents could positively influence the drug retention rate and the possibility to stop GC treatment.

## Data Availability

Data is provided within the manuscript.
